# Assessing community (peer) researcher’s experiences with conducting spirometry and being engaged in the ‘Participatory Research in Ottawa: Management and Point-of-care for Tobacco-dependence’ (PROMPT) project

**DOI:** 10.1186/s40900-018-0125-z

**Published:** 2018-12-01

**Authors:** Catherine B. Charron, Alzahra Hudani, Tina Kaur, Tiffany Rose, Kelly Florence, Sadia Jama, Smita Pakhalé

**Affiliations:** 1 University of British Columbia, Vancouver, Canada; 2 University of Ottawa, Ottawa, Canada; 3 University of Toronto, Toronto, Canada; 4 The Bridge Engagement Centre, Ottawa, Canada; 5 Ottawa Hospital Research Institute, Ottawa, Canada; 6 Division of Respiratory Medicine, The Ottawa Hospital, 501 Smyth Road, Ottawa, K1H 8L6 Canada

**Keywords:** Community researchers, Community-based research, Patient engagement, Tobacco, Smoking cessation, Spirometry training, Power differential

## Abstract

**Plain summary:**

This article examines the overall experiences of community researchers in their involvement with the ‘PROMPT’ project for smoking cessation, which targeted community members who were homeless or at-risk for homelessness. More specifically, four community members, representing the study population were involved in the project as researchers. They were asked to complete surveys at both the beginning and end of each research training session to better understand their learning as it related to using a key instrument for this project, a spirometer, to measure project participants’ lung function. Spirometry is typically performed by trained healthcare providers. Community researchers were also interviewed to explore what their experiences were like working as a researcher with their own at-risk community. Although the researchers felt that the training was sufficient, more research is needed to evaluate training effectiveness among community researchers in delivering acceptable quality lung function testing using a spirometer. Upon analyzing the small group discussion and survey results, we found that the community researchers had an overall positive experience with both the project, and the training that was provided to equip them with the knowledge, tools, and resources they needed to successfully work in a research project of this kind. They also faced challenges that are common in such community-based projects, such as the power differential between the researchers with a healthcare background and themselves who have lived experience with the issue at hand.

**Abstract:**

**Background**

The Ottawa Citizen Engagement and Action Model (OCEAM) used a Community Based Participatory Action Research (CBPAR) approach by involving the most at-risk urban population. Community (peer) researchers participated in every step of the study despite the multiple challenges.

**Objective**

To assess the community researchers’ training and experiences in a CBPAR project, PROMPT: Participatory Research in Ottawa: Management and Point-of-care for Tobacco Dependence.

**Method**

Four community researchers were recruited, representative of the PROMPT project’s target population with current or past poly-substance use; smoking tobacco; and/or being homeless or at-risk for homelessness. The community researchers participated in all phases of PROMPT, including study design, development of questionnaires, participant recruitment, administering consent forms and questionnaires, as well as hand-held spirometry after rigorous training. To assess their knowledge and comfort level with spirometry testing after standardized training, questionnaires were administered pre- and post-training. In turn, to assess their overall experience, interviews were conducted at the end of study completion.

**Results**

All community researchers underwent small-group training sessions including presentations, discussions and hands-on practice adapted from standardized training material prepared for health care professionals. Spirometry training was included in all sessions. Self-perceived knowledge and confidence in administering spirometry, as well as skill-testing score averages improved between the pre- and post-training questionnaires. Overall, all the community researchers had a fulfilling experience participating in the project.

**Conclusion**

Despite challenges, involving community researchers with lived experience is feasible, satisfying and productive even in the most marginalized populations. Standardized spirometry training of community researchers’ representative of the PROMPT target population, with no healthcare educational background, was feasible and effective in improving knowledge, confidence and readiness to administer spirometry.

## Background

Patient engagement in research is upheld by all healthcare funding agencies in the USA, Canada and globally [1–3]. However, meaningful engagement of patients in healthcare and research remains scarce and tokenism remains prevalent [4]. Involving patients in research has many challenges; it is time consuming and labour intensive. Involving the most marginalized, homeless, at-risk for homelessness and low-income populations in research brings another layer of challenges due to the many social, political, structural, environmental, and economic determinants of health faced by them. In the Community-Based Participatory Action Research (CBPAR) approach, community members are involved in all research phases. This framework presents multiple advantages such as improving the health outcomes and reducing social disparities particularly in low socio-economic populations. However, few studies have looked at the community (peer) researchers’ experience when utilizing this approach. Moreover, the use of community involvement to ensure equity, respect and improve health outcomes is still unclear.

Studies of most at-risk inner city populations such as People Who Use Drugs (PWUD), homeless and at-risk for homelessness populations are limited [5]. Yet, PWUD utilize significantly more acute healthcare services such as emergency department visits and require frequent hospitalization when compared to non-illicit drug users [6]. In an American study, rates of hospitalization were four times greater in the homeless population than the general population [7]. This suggests that there is a lack of appropriate outpatient services and a delay in this population seeking necessary medical care. Multiple factors limiting outpatient visits have been identified in the most at-risk communities of PWUD, including the lack of trust in health care providers, lack of case managers, fear of discrimination, costs associated with the visits to a healthcare facility and the lack of health insurance [8, 9]. Community-based participatory interventions and research may be able to overcome some of these barriers.

Importantly, it is well known that the prevalence of tobacco smoking is significantly higher in PWUD and in the homeless or at-risk for homelessness populations [10–12]. However, the diagnosis and management of tobacco smoking related diseases are often limited in this population. Undoubtedly, these marginalized communities would benefit from adequate and consistent follow-up and care including spirometry testing to diagnose diseases such as Chronic Obstructive Pulmonary Disease (COPD) and asthma, within or as a component of smoking cessation interventions. In fact, death rates related to tobacco use were greater than death rates related to other substance use such as alcohol, which was the substance more prevalently used in the study by Hurt et al. [13]. Spirometry is a recommended test to assess pulmonary function and to diagnose diseases such as asthma and smoking related lung diseases such as Chronic Obstructive Pulmonary Disease (COPD) [14]. It is an office based device which requires a forceful inspiration and exhalation through a mouthpiece which provides quantitative information on lung functioning. Despite the strong recommendations of requiring spirometry to make the diagnosis and manage diseases such as COPD and asthma, the utilization of spirometry for this purpose is quite poor [14–16].

In community based participatory interventions and research, a partnership is formed between community members and healthcare professionals or research scientists. The term participatory research has been defined as research involving those affected by the issue being studied [17, 18]. The community based participatory approach has many reported advantages. For example, collaborating with community members with lived experience ensures that the research question is tailored to the community’s needs; and that the intervention is culturally appropriate and aims at creating a lasting impact [19–21]. Most importantly, this approach has been shown to reduce social disparities, particularly in low socio-economic populations [22]. Community based participatory interventions have been adopted and studied mainly in health promotion. For example, in HIV prevention models, community members such as those who are current or past illicit substance users, deliver interventions that would have classically been delivered by a health care professional [23]. Methodology of community-based participatory research is not yet well understood in areas such as the allocation of tasks between community members and academicians. The Ottawa Citizen Engagement and Action Model (OCEAM) uses a Community Based Participatory Action Research (CBPAR) approach by involving the most at-risk urban population [18]. To maximize the efficiency of research steps and health outcomes, further research on the use of CBPAR principles is necessary and long overdue [21].

Although some community based participatory research has been conducted in tobacco smoking dependence [24], community members have never been involved in the process from end-to-end, especially in data collection or the administration of lung function testing. The PROMPT project, Participatory Research in Ottawa: Management and Point-of-Care for Tobacco Dependence, was designed to assess tobacco dependence management strategies in the most at-risk inner city population with a Community-Based Participatory Action approach in true partnership with the target population. Additional details on the PROMPT project protocol are described in a previous publication [18].

The objective of this study is to assess the community researchers’ experiences with spirometry training and their overall experiences participating in a CBPAR project, PROMPT: Participatory Research in Ottawa: Management and Point-of-care for Tobacco Dependence. The aim was also to assess the experiences of community researchers who had no educational background in healthcare, or in administering spirometry testing.

## Methods

In the PROMPT project, four community researchers were selected from the study target population comprised of current or previous tobacco and poly-substance users, who were homeless or at-risk for homelessness and resided in the inner-city region of Ottawa. The community researchers were recruited by word of mouth and selected after interviews. Candidates were selected based on their keen interest in developing research in their community, previous experience as well as communication and networking skills. This study was approved by the Ottawa Health Science Network Research Ethics Board, and written informed consent was obtained from all participants.

**Fig. 1:**
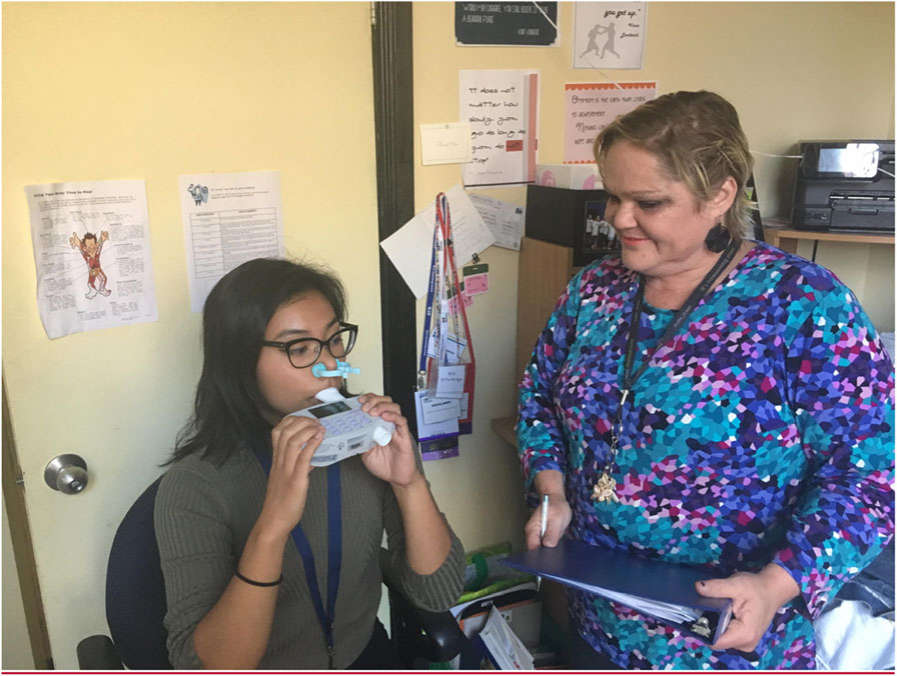
Spirometry being performed at the Bridge Engagement Centre

Community researcher training focused on confidentiality, privacy, the Tri-Council Policy Statement-2 [25], verbal and non-verbal communication, diversity of the study participants, general ethical concerns in research with respect to working with the most at-risk populations, up-to-date health disparity literature, inclusivity and conflict resolution. The community researchers underwent training to obtain participants’ consent and to administer the study questionnaires. In addition, they underwent standardized hand-held spirometry training and certification as per the Burden of Obstructive Lung Disease - BOLD study [15] and the Canadian Cohort of Obstructive Lung Disease - CanCOLD study spirometry training guidelines [26]. Although standardized training material was used, the vocabulary was adapted in order to be understood by the community researchers, as they did not have a background in healthcare. The project and training was implemented at the community research office, The Bridge Engagement Centre (The Bridge), located in downtown Ottawa, where the majority of Ottawa’s most at-risk PWUD, homeless or at-risk for homelessness populations are concentrated. The six-day training was led by a respirologist (SP) trained in epidemiology, pulmonary function testing and interpretation. Spirometry was performed with the ‘EasyOne’ hand-held diagnostic spirometer from NDD Medical Technologies, as per the guidelines [15, 26] (See Fig. 1). To ensure the quality of the spirometry test, community researchers were required to achieve at least 8 manoeuvres of grade A or B programmed by the hand-held spirometer [15].

Community researchers’ feelings of safety and comfort were at the core of all training sessions. Training was conducted in a low-threshold and non-judgmental fashion at The Bridge. The training was organized in a small group with the four community researchers and was adapted to their level of knowledge, comfort and education level. The group sessions included didactic presentations on research concepts, lung physiology and spirometry, discussions and hands-on practice with administration of consent, surveys and the hand-held spirometer. Community researchers were encouraged to ask questions and open topics of discussion with the team during the training session. To facilitate this open environment into the training session, frequent breaks were planned into the schedule, to include opportunities for community researchers to ask questions.

Community researchers filled out a short questionnaire before and after each of the six training sessions. Training sessions included a single one-on-one, and six small-group training sessions over the period of 4 weeks. A total of 19 questionnaires were completed: 9 before and 10 after completion of the training sessions. The items on the questionnaire (Appendix A: Table 1) attempted to measure the following primary outcomes: community researchers’ overall satisfaction and experience with the project training, self-reported knowledge of lung function, confidence in administering spirometry, and objective knowledge which was assessed by skill-testing questions. Recommendations for changes in the training process were also sought with an open-ended question at the end of the questionnaire. As per the PROMPT project procedures, community researchers were paid an honorarium of $15 per hour for attending all training sessions, for a total of 48 h.

The community researchers participated in all research phases of the PROMPT project, which consisted of: conception of the research question, designing questionnaires for program implementation as well as knowledge creation, translation and mobilization (data cleaning, analysis, abstracts, posters, manuscript writing and presentations). The majority of research activities including participant enrollment, obtaining consent, administering surveys, testing, follow-up visits, and manuscript writing took place at the Bridge Engagement Centre, where team members worked hand in hand. The community researchers recruited and enrolled a total of 80 PROMPT participants and were responsible for obtaining consent, initial intake survey administration, administering spirometry testing and monthly follow-ups. A nurse and a research coordinator were on site during spirometry testing to provide assistance in case of an adverse reaction. Recruitment and retention strategies designed by the community researchers were implemented, with an emphasis on social-network based approaches. An interview with the two most involved community researchers was held after completion of the PROMPT project to learn about their experience and to receive feedback about the project. Open ended questions were asked in relation to the community researchers’ participation in the project in the following three categories: 1) personal gains and challenges with project leadership, 2) perceived gains and challenges faced by the community and participants, and 3) insights about overall project successes and challenges. The interview was audio recorded, transcribed and reorganized into the three categories.

## Results

The demographic information of the community researchers is specified in Table 2 (Appendix B). Three of the four community researchers were male, and between the ages of 40–50; and one of the researchers was between 30-40 years of age. Their level of education varied from having a high school diploma to some college or university education. None of the community researchers’ had a background in healthcare or health education. All participants completed the project training and spirometry certification successfully. Below is the community researchers’ self-reported knowledge assessment for the spirometry training they underwent, as well as their overall experience in their role within the PROMPT project.

### PART 1: Self-reported knowledge assessment

**Fig. 2:**
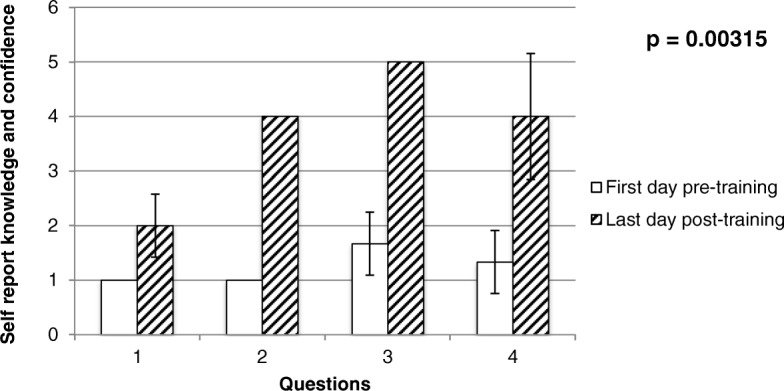
First day pre-training and last day post-training self reported knowledge of lung function, spirometry and confidence in administering spirometry

The following section is an analysis of the results from the completed pre- and post-training questionnaires that were administered. Responses to questions about self-reported knowledge and confidence in administering hand-held spirometry tests illustrates that participants had minimal knowledge of the anatomy and physiology of the respiratory system prior to the training. An improvement in both self-perceived knowledge and confidence in administering hand-held spirometry was observed when comparing participants’ responses before and after the entire training program (See Fig. 2). Importantly, after completion of the final training session, the participants felt very confident in being able to administer a good quality lung function test. Community researchers indicated, on their first pre-training workshop questionnaire, that they believed further training would help improve the quality of the spirometry result. However, upon completion of the training, in the final questionnaire, they disagreed that they needed further training.

**Fig. 3:**
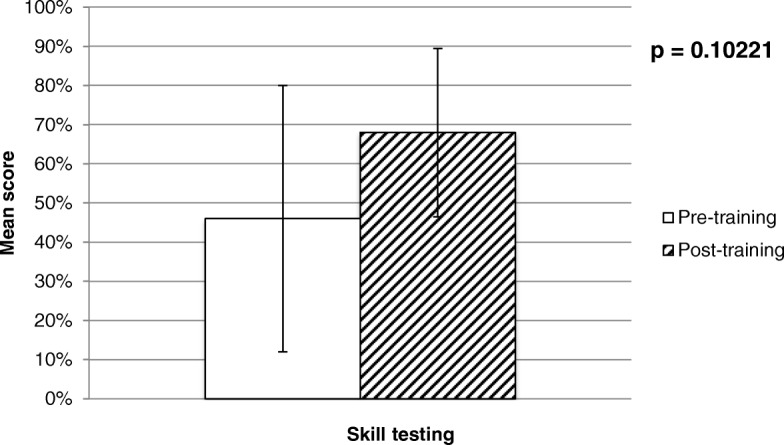
Pre- and post-training skill questions’ mean

When comparing the mean of the answers to the pre-and post-training session skill-testing questions asked on each day of the training, there was no significant improvement as shown below in Fig. 3.

**Fig. 4:**
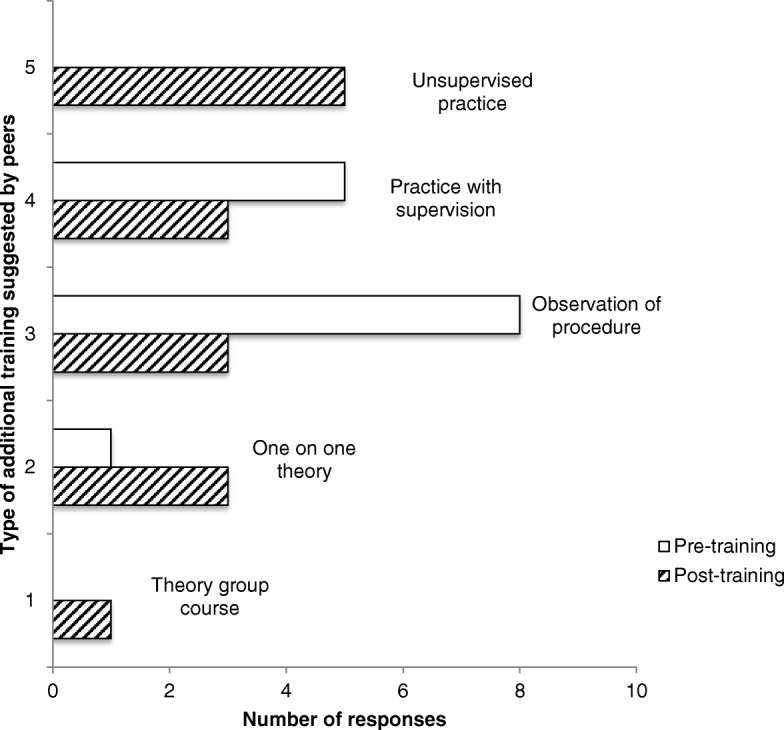
Pre- and post-spirometry training preferences in training type

The type of training they would most benefit from varied considerably amongst the community researchers. In the pre-training workshop questionnaires, community researchers indicated a preference to observe the procedure performed by the trainer, while unsupervised practice was selected more often by the community researchers in the post-training workshop questionnaires (See Fig. 4). Post-training questionnaires demonstrated that the training workshops were very helpful. Community researchers did not provide any constructive feedback about the training on the open-ended survey question listed in the post-training questionnaires.

### PART 2: Evaluating community researchers’ overall experience

The following is an analysis of the results from the interview at the end of the study with the two most involved community researchers to understand their: 1) personal gains and challenges with project leadership, 2) perceived gains and challenges faced by the community and participants, and 3) insights about overall project successes and challenges.

I. **Personal Gains & Challenges with Project Leadership**

The first part of the questionnaire focused on the community researchers’ personal gains achieved and challenges faced through participation in the study. The major themes that emerged as personal gains included: an increase in self-confidence; feeling like valuable members of the community in their role as community researchers; an increase in knowledge of both research methods and smoking physiology; and feeling motivated for self-change. The community researchers stated that the experience provided, “*inspiration to continue to find ways to help others*,” and “*motivation for self change through a safe space for sharing and seeing others having success with their own changes* [with tobacco smoking].” They also stated a feeling of “*increased respect for other members of the community*,” and gratitude for having the “*opportunity to grow and learn by participating in all research steps such as data analysis and grant submission*.” The increase in confidence was stated as a result of experiencing “*high levels of trust, respect, and responsibilities*.” In fact, the community researchers found that the “*project never ends at the door as a community peer*,” as study participants were contacting them even after hours, further demonstrating the importance of their role in directly interacting with the study participants and in leading a project of this kind. However, there were also some challenges, especially around the usual challenges that occur when implementing a community engagement framework. Power issues between the academic researchers and community researchers are real and many a time unavoidable [27]. Throughout the process, community researchers expressed feeling a power imbalance between the academic researchers on the team and themselves. A community-researcher expressed, “*At times I felt like there was a hierarchy and felt that I was not heard. I felt that since I did not have as much education as others my contribution was not as valued*.”

Navigating issues around power were challenging for both the academic researchers and community members. The delicate balance between day-to-day challenges experienced by project participants and the demands of the rigorous research protocol at times led to feelings of tension, underpinned by the experience of a power differential between members of the research team. For example, it was challenging on occasion to negotiate the follow-up schedule mandated by the research protocol and the accommodation of various needs and challenges faced by community researchers and participants on access to transportation, legal issues and/or infrequent and unreliable communication. However, community researchers had an overall, very positive experience participating in this project.

II. **Perception of Community Gains & Challenges**

The second part of this questionnaire assessed the community gains and challenges that were experienced and/or observed by the community researchers. The community researchers had not expected that as many of the study participants would reduce or quit tobacco use as they in fact did. They were also surprised to observe that this change in tobacco smoking among study participants impacted several of the other determinants of health, which then positively impacted both their lives and health. They noted that this commitment to reduce smoking was an accomplishment in itself, and was associated with building confidence within the community, and was also a source of motivation among the group. Additionally, a community researcher commented, “*When people stopped coming, I thought they might be out partying when in fact some were getting jobs, going to rehab, or in the hospital getting better. At times, we stigmatize our own community and ourselves.*” This comment provides valuable insight not only on the positive changes that participants were making for the betterment of their health and quality of life, but also the problematic and harmful misunderstandings, perceptions, and stigma that can be associated with their actions.

Furthermore, community researchers felt that having a limited capacity for the PROMPT intervention was a factor that caused division in the community. Overall, we learned that while implementing the project with a shoestring budget itself is challenging, implementing it in a significantly marginalized population adds another layer of threat due to the challenges they face. Therefore, not having enough funding to cover all costs and enroll more participants after the project led to traction in the local community, and was difficult at best. Community researchers felt that the project taught them a great deal personally and communally.

III. **Community Researchers' Insights about Project Success & Challenges**

Intervening in the lives of participants who smoked and came from at-risk communities initially led to perceived challenges of program inertia, and uncertainty about project success. Community researchers indicated that the resistance to make changes to the program was influenced by the need to maintain trust among study participants, as this community “*often has trust issues from past experiences*.” Secondly, uncertainty with respect to program success was also identified as a challenge as community researchers felt accountable as program leaders. They expressed that, “*service providers can just go back to the office, but as a peer you can’t hide from your own community*.” In addition to these perceived and actual challenges, came perceived and real successes. Community researchers found that participants were motivated and therefore easy to recruit; that front line health professionals involved in the study were able to provide support that extended beyond smoking cessation; and that the OCEAM, which was achieved through the PROMPT program, is a framework that they believe will impact the way others studies are run. A community researcher stated, “*Many people are interested in integrating elements of our project into their own. I hope we can show people it works, and the snowball effect that will happen from it…I have faith that this will turn into a model that others will use*.” This statement demonstrates that the community researchers were pleasantly surprised by the project’s outcomes and benefits gained by their community. They truly believe that other research teams working in various settings could adapt this community engagement model into their diverse programs.

Overall, the community researchers reported regaining confidence in the professional community, and provided constructive feedback on the project processes. In addition, they indicated a gain in self-confidence, feelings of inspiration, and a gain in knowledge and skills through their active participation in the project. PROMPT project outcomes unexpectedly impressed the community researchers, as participants not only reduced and/or quit tobacco smoking, but also other illicit substances such as fentanyl and oxycontin. Furthermore, community researchers were pleasantly surprised to observe that many of the PROMPT participants made additional improvements in other areas of their life; such as making the decision to return to school or work, eating healthier, and engaging in community-based work [12]. More specifically, the feasibility study demonstrated that of the 80 participants recruited in this study, 79% reported reducing their tobacco intake, 9% reported quitting tobacco use, 18.8% reported reducing poly-substance use, and 30% improved their general socio-economic status gradually helping them to improve their health and quality of life [12].

## Discussion

This study has demonstrated that CBPAR, with a true partnership between community researchers and academicians throughout the research process, is feasible and more importantly mutually beneficial. In the PROMPT project, it is clear that community researchers were essential to the project’s design, implementation and success. Community researchers gained self-confidence, motivation, knowledge and skills through their active participation in the project. Furthermore, various tools to measure the effectiveness of patient engagement strategies are emerging and available. For example, the Centre of Excellence on Partnership with Patients and the Public developed an evaluation toolkit to improve engagement at all levels of the health system, whether it be in health research or healthcare. The toolkit consists of 27 distinct evaluation tools, which are sorted for the organization, project, or participant levels [28]. Moreover, patient engagement can improve patients’ knowledge and experience, use of health services, health behaviour, and health status [29]. The PROMPT project demonstrated that health behavior and health status of community researchers and the majority of the project participants did improve [12]. However, involving patients can be challenging if one considers multiple goals of care for an individual [30]. Setting priorities and defining goals in partnership with patients could mean that researchers might have to adapt and compromise some of their own research aims. Research methods may need to evolve to suit the community researchers’, as well as community and participants’ goals. This evolution might bring challenges around power and decision-making [27]. This project did bring out some of these challenges as demonstrated in the discussion held at the end of project with the community researchers, who reported sensing a hierarchy and resistance from academic researchers to evolving change in order to maintain scientific rigor.

Standardized spirometry training of community researchers’ representative of the PROMPT project target population, with no healthcare educational background, was effective in increasing knowledge, confidence and self-reported readiness to administer spirometry. To the best of our knowledge, this is the first study on spirometry training for community researchers who do not have a healthcare background. Community Based Participatory Action Research (CBPAR) though gaining popularity, is still rare. Ideally in CBPAR, community members should participate in all phases of a research study. In the PROMPT project, community researchers were involved throughout the project, from formulating the study question to knowledge mobilization through the Ottawa Citizen Engagement and Action Model (OCEAM) [18]. The community researchers demonstrated exceptional leadership, especially in maintaining confidentiality and privacy with respect to the research process and participant information. The CBPAR framework presents multiple advantages and challenges. Equitable partnership between community members and academics is beneficial [20] but specific means to achieve this and optimize outcomes still remain unclear [31].

The quality of spirometry test results depends on several elements such as the participant’s effort and cooperation, underlying lung disease severity, the equipment, and the technicians. More specifically, spirometry standardization requires the technicians to provide appropriate instructions and corrections [32]. The most common issue encountered during lung function testing is an insufficient respiratory effort. Technicians need to encourage patients to continue forceful exhalation during spirometry and the manoeuvre is repeated until it meets criteria. A CanCOLD sub-study has shown that technicians’ prior experience did not affect the quality of spirometry testing as long as they received standardized training [26]. However, that study was performed with health care workers that supposedly had a basic knowledge of human physiology and lung function. The PROMPT project was unique, in that the community researchers were selected from the study target population, which is an essential element of the community based participatory approach. They were also not required to have any prior health care training, and were therefore specifically trained to administer spirometry for the PROMPT project. None of the community researchers had significant background, knowledge, training or education on basic knowledge of human physiology and lung function, or healthcare education. Although the community researchers had limited knowledge of the anatomy and physiology of the respiratory system and of spirometry testing prior to the training, the primary outcomes, perceived knowledge and confidence in administering spirometry, improved after training. Additionally, there was self-reported readiness to administer acceptable quality spirometry testing after completion of the training.

The pre- and post-spirometry questionnaire analysis has several limitations that could attribute for the lack of significance of average scores on the pre- and post-training skill-testing questions. First, the study group was small. Also, averages of the scores on the pre- and post-training questionnaires were similar. This is potentially due to the fact that questionnaires were administered twice daily, before and after the training sessions. Results of post-training questionnaire scores were similar to the following day’s pre-training since no activities related to the study occurred between them. In addition, by repeating questionnaires, the community researchers likely had remembered the skill-testing questions. Such regular repetition could have biased the results. Perhaps the questionnaires should have been administered only at the very beginning and at the very end of the entire training. There was high satisfaction with training and there were no recommendations for changes in our training process. If modifications were to be made to the training it would not be based on these results. Though we used standardized CanCOLD spirometry training [26], we simplified the language to ensure it was accessible to the community researchers, as they did not have a background in healthcare. In addition, we attempted to adapt the training by administering it in interactive, small-group teaching sessions. This allowed time for questions, repetition and ample hands-on experience. When creating or modifying training programs for community researchers, knowledge and skill building should be the focus, with a greater proportion of the training dedicated to hands-on experience, the preferred learning method for adults [33]. The Guidelines for Practice and Training of Peer Support also suggest that adapted scheduling and accessibility are important considerations for such training programs [33].

For the training and PROMPT project protocol, the ‘EasyOne’ hand-held spirometer was used ensuring quality of the test with its integrated quality control. Although our results show that community researchers felt the training was sufficient, further research is required to assess community researchers’ effectiveness in implementing acceptable quality spirometry long term. Implementation of this training protocol for community researchers will save resources for the general population and encourage enrolment of vulnerable and the most at-risk populations in future research studies. This could also be a model used in the community to increase access to outpatient medical services, as spirometry is not adequately used in the community to diagnose diseases such as asthma and COPD [34, 35]. Importantly, most people with COPD in Canada remain undiagnosed; and the overall health system burden caused by exacerbations in those with undiagnosed COPD is considerable [36]. Community research support fosters hope and motivation to change, in addition to facilitating access to vital health care services. In fact, community supported recovery has been demonstrated to hold promise for improving clinical outcomes [37].

## Conclusion

Health promotion through community-based interventions is a promising approach to address health disparities of the most at-risk populations by providing care that is responsive to the needs and circumstances of patients. The development of tailored and culturally sensitive training programs is feasible and requires further patient and community engagement. This project demonstrated that community members with lived experience, who represent the PROMPT target population, could be successfully engaged and trained in project design and implementation, including administering tests and procedures such as hand-held spirometry. Hand-held spirometry training as per CanCOLD’s guidelines, for non-healthcare, spirometry naϊve community researchers, was feasible and effective in improving knowledge, confidence and self-reported readiness to administer hand-held spirometry in a CBPAR project. Implementation of the study’s training protocol for community researchers would save health care resources and encourage enrolment of vulnerable populations in future research studies. Research is still required to assess community researchers’ effectiveness in implementing acceptable quality spirometry testing over time.

## Appendix

**Table 1:** Pre- and Post-training workshop questionnaire

A. Self-reported knowledge and confidence in administering spirometry test	
1. How much do you know about the anatomy and function of the respiratory system? 2. How would you describe your knowledge of the lung function tests? 3. How confident are you that you can administer and obtain a good quality lung function test with your current knowledge and abilities? 4. Do you believe training or further training in lung function testing would help you improve the quality of the test result?	
B. Skill-testing questions	
1. What does FEV_1_ stand for? a) The forced expiratory flow over 1 min b) The total volume of air exhaled in a forced expiratory manoeuvre c) The amount of air that a person breathes out during the first second of a forced expiratory manoeuvre d) I don’t know	
2. A good lung function test is 3 manoeuvres that have no mistakes and that have similar results, how many times should you repeat the test until you obtain a good result? a) Once b) Up to 15 min c) 3–5 times d) Up to a total of 8 times assuming the subject is able to continue e) I don’t know	
3. Select as many of the following situations in which you would not perform a lung function test to a patient with: a) Stroke or heart attack in past 3 months b) Drug or tobacco use in past 3 months c) Chest or abdominal surgery in past 3 months d) Eye surgery or detached retina in past 3 months e) Tuberculosis currently treated with medication	
4. Select as many of the situations that could cause an error in lung function testing: a) Hesitation or false start of expiration b) Cough c) Leaks (participant not able to keep at tight seal between lips and mouthpiece) d) Stopping expiration early e) Inadequate coaching or directions to participants	
C. Usefulness of training workshop	
1. What type of training or practice could you benefit more from? a) Group lecture or theory type course b) One on one theory course c) Observation of the procedure done by a trainer d) Practice with a coach who provides feedback e) Unsupervised practice 2. Was the training workshop helpful? 3. Comments, questions or suggestions	

### Appendix

**Table 2:** Community (peer) researcher demographics (*n* = 4)

Identification	
Age	3 (40–50): 1 (30–40)
Gender	3 M: 1 F
Level of education	2 High school diploma: 2 with some college or university studies
Employment	2 Part-time: 2 student or in training
